# Magnetic oriented microparticles preparation

**DOI:** 10.1016/j.mex.2020.100975

**Published:** 2020-06-21

**Authors:** Tzuriel S. Metzger, Avi Schneider, Naama Goren, Amir Ziv, Yair Tocatly, Eytan Avigad, Shira Yochelis, Yossi Paltiel

**Affiliations:** Dept. of Applied Physics, Hebrew University, Jerusalem, Israel

**Keywords:** Janus particles, Magnetic particles, CISS effect, Spin controlled enantiomer separation

## Abstract

Generally speaking, reaction platforms involving ferromagnetic surfaces, with a specific magnetic direction, are limited to the two dimensional regime, due to the nature of the magnetic phenomena. Here we show a method for preparing partially coated ferromagnetic microparticles with a distinct magnetic pole. This simple preparation method was presented previously ^[^^1^^]^ to demonstrate an application for enantiomeric separation.

In this method article we show;

• A simple method to a-symmetrically manipulate particle surfaces.
• A generic way to synchronize a bare pole of ferromagnetic microparticles.
• A simple and generic enantiomer purification technique.

## Graphical abstract

**Figure:**
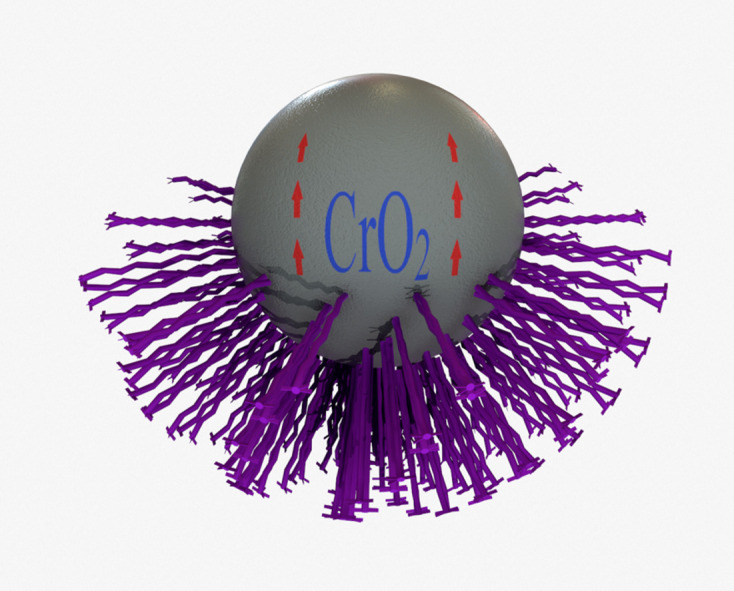

**Table:** Specifications Table

Subject area:	Materials Science
More specific subject area:	Magnetic particles, manipulation on functionalized particles
Method name:	Magnetic Oriented Microparticles Preparation
Name and reference of original method:	N.A.
Resource availability:	Particle source, (CFM-1000–5, CFM-40–10):
	https://www.spherotech.com/2016%20Catalog%20Product%20Detail%20Pages/Spherotech %20Ferromagnetic%20Particles.pdf

## Method details

### A-symmetric manipulation of particle surfaces

1. ***Starting with symmetric particles*** - The process begins with a 1% w/v aqueous suspension of polystyrene-chromeoxide core-shell microparticles, coated with 11-carbons-long hydrocarbon ligands with a terminal carboxyl group (Spherotec @ CFM-1000–5, 102 µm diameter, or Spherotec @CFM-40–10, 4.67 µm diameter).
2. ***Breaking the symmetry using a glass substrate*** - The particles are dripped onto a glass substrate (Fig. 1A). This process is critical for breaking the symmetry by having one side of the particles protected by the glass surface.

**Fig. 1:**
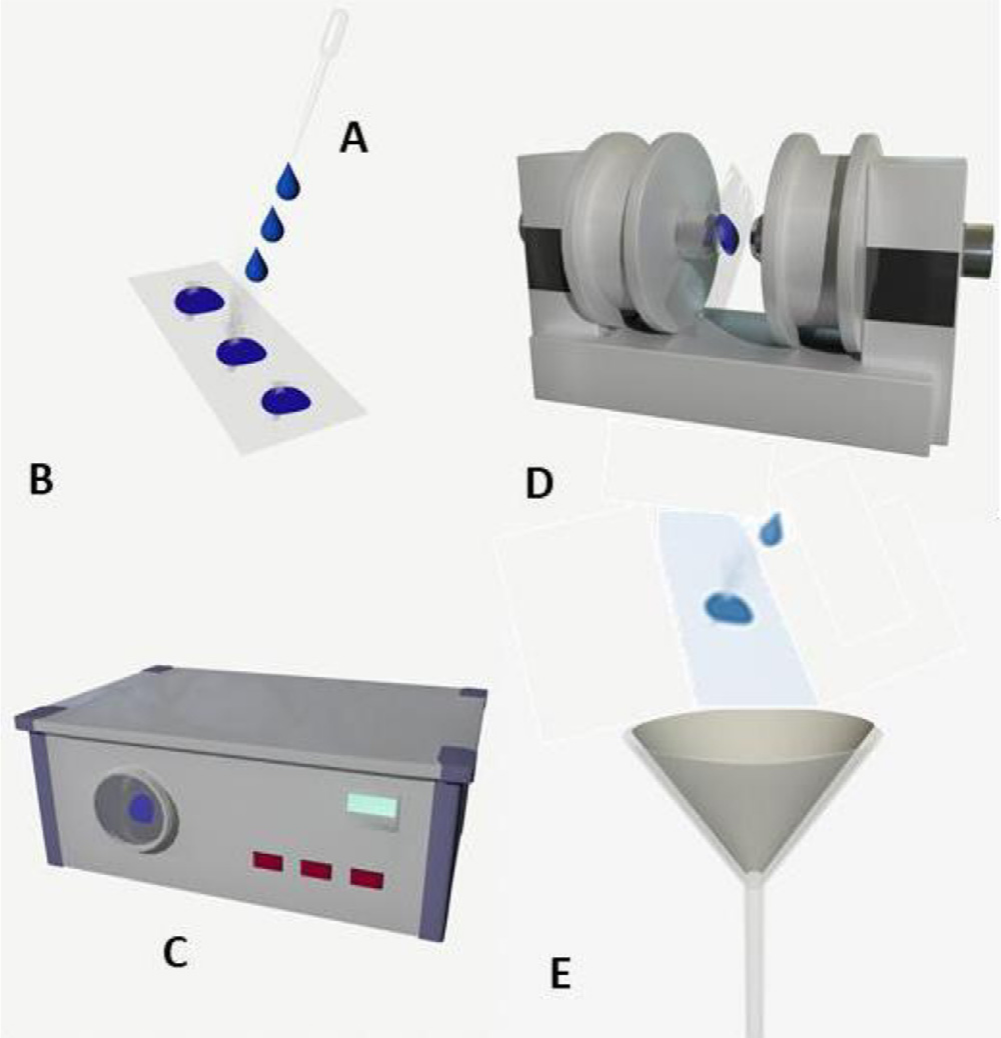
Scheme of the entire process; A-B. deposition and drying of the microparticles on a glass substrate, C. 10 min treatment inside the plasma asher device, D. Magnetization under 2500 Oersted magnetic field (electromagnet), E. Washing and collecting of the microparticles into a filter paper.
3. ***Drying the sample*** - The glass substrate with the microparticles is dried over a hot plate to form a granular film with a radius of about 1 cm (Fig. 1B). The procedure requires pre-heating the glass substrate (2.5 * 7.5 cm) on a hot plate to about 60 °C. After a few minutes of pre-heating, drops from the microparticle suspension are placed at equal distances to generate a granular film that is uniformly spread. This process is done multiple times on different areas of the glass substrate. Once the solvent from one drop is evaporated, another drop of the particle suspension is placed on the glass substrate. The distance between drops should allow for easy washing of the particles at the final stage.
4. ***Creating the Janus particles*** -The particle-coated glass is placed in a plasma asher (Diener PICO UHP). The device is set to 50% power (100 W) for 10 min (Fig. 1C), using a 50% oxygen mixture, in order to create oxygen plasma with the ability to etch off the organic molecules exposed to it (facing up). At the end of this stage, only the portion of the microparticles’ surface which faced upward, and was exposed to the plasma, is ligand free, while the portionprotected by proximity to the glass, remains covered withligands.

### Synchronizing the microparticles' magnetization

5. ***Magnetization of the micro particles*** - The fixed orientation of the microparticles magnetization is achieved by applying an external magnetic field. The dried microparticles on the glass substrate are placed (Fig. 1D) in a perpendicular magnetic field of about 2500 Oersted produced by an electromagnet.
  This process orients the magnetization of all micro particles. The breaking of the symmetry is a result of the orientation of the magnetic field that aligns the ferromagnetic spins on the half of the particle covered with an organic ligand layer. The non-covered area of all the particles is now magnetized in the same direction. A schematic diagram of the final particle is shown in Fig. 2, after applying a magnetic field in the direction of its exposed half.

**Fig. 2:**
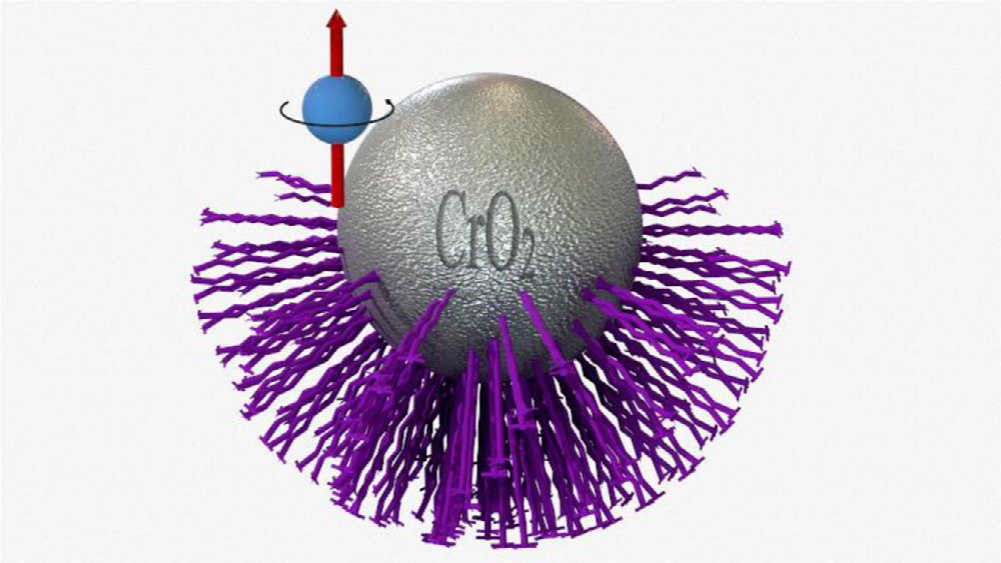
Scheme of the final particle after applying a magnetic field. The chart is not to scale; the ligands are represented in the purple layer. The spin direction is defined by the external induction of the electromagnetic field after the plasma operation.
6. ***Washing and collecting the Janus particles***- The microparticles are washed away from the glass substrate into a filter paper. The particles are then collected from the filter (Fig. 1E).

#### Characterization

**Fig. 3:**
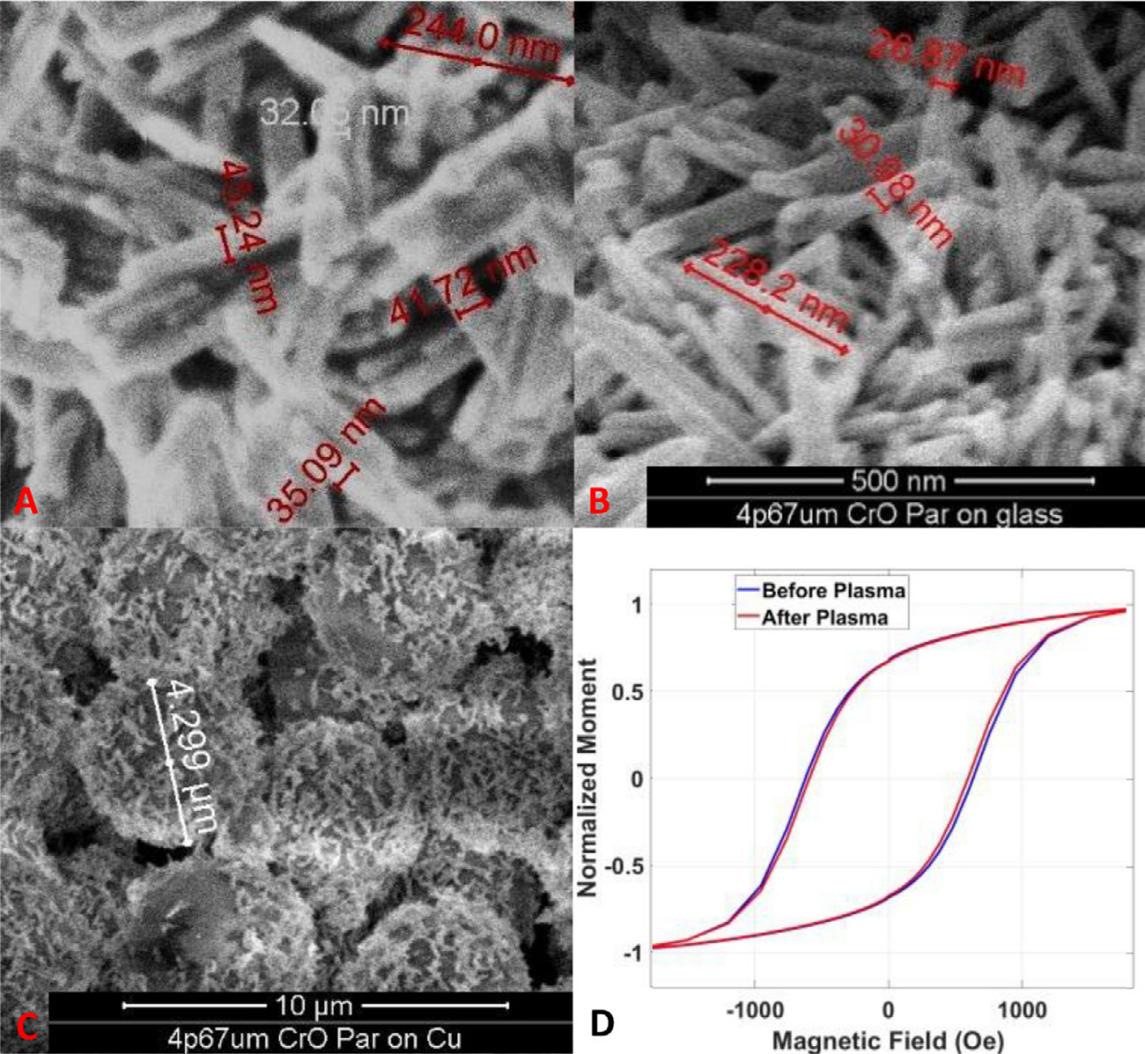
MagellanTM 400 L SEM images of the Chromium oxide rods, which comprise the shell of the microparticle, covering its polystyrene core, before A) and after B) the plasma asher process. C) A wide-angle perspective of whole microparticles. D) Magnetic hysteresis loop of the particles measured using a superconducting quantum interference device (SQUID), before and after the plasma asher process. In this measurement the sample was kept at room temperature.

Fig. 3A-C presents SEM images of these core-shell microparticles before and after the plasma asher process. Fig. 3D shows a magnetization hysteresis loop of the particles. As can be seen, aside from removing the ligands (are not visible in the SEM image), the plasma treatment has no recognizable influence on the shape, surface morphology or magnetic properties of the microparticles. The particles remain hard ferromagnets with a saturation field of about 2000 Oersted - which does not change during the plasma process, assuring that the plasma did not damage the particles. These hysteresis loops measured with sample chamber at room temperature. It is important to emphasize that the plasma asher of the particles on a magnetic surface, enable to synchronized the particles magnetization at room temperature and the microparticles exposed surface directions creating selective an interaction surface that is enantiomer selective.

**Fig. 4:**
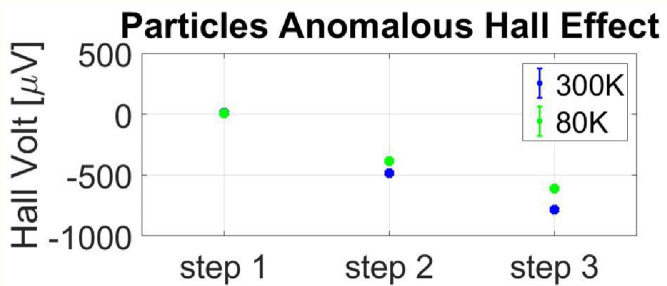
Anomalous Hall effect of the particles before (step 2) and after (step 3) palsma, at RT (blue) and at liquid N_2_ temperature (green). Measurements of the Hall effect under 0.5 T give similar results.

To further characterize the magnetization of the particles, we have measured the magnetization of the particles monolayer at different preparation stages using a Hall device. Device of a 5 * 5 mm thin layer of gold was prepared, with a covalent bonded monolayer of chromium oxide particles. The Hall voltage is measured and averaged between four directions of current / voltage, relative to the clean gold layer. Fig. 4 compares the particle induced Hall voltage at 80 K and 300 K for three preparation steps. Step 2 is the monolayer formation of the particles; Step 3 is the 10 minutes’ plasma irradiation. These measurements were done with and without a magnetic field (anomalous Hall effect). Indeed, small changes in magnetization were measured after the plasma treatment.

To assure a non-symmetric ligand coverage of the microparticles, contact angle measurements were conducted. Fourier Transform Infrared spectroscopy (FTIR) measurements were not sensitive enough to measure the change in coverage. The contact angle measurements enabled a more sensitive probing of the plasma treatment's effect on the surface of the particles. The plasma time was calibrated to remove the ligands from the exposed (top) hemisphere of the particles without harming those on the glass-facing (bottom) side. This calibration is critical and changes with the use of different particles. The contact angle measurements were performed on three types of samples: untreated particles, particles exposed to plasma, and particles exposed to plasma which were then mixed and redeposited at random orientation. Each sample was measured at two distinct areas: the center of the microparticle film - a region comprised of microparticle multilayers, and at the edge of the film - a region approximately representing a single microparticle layer.

**Fig. 5:**
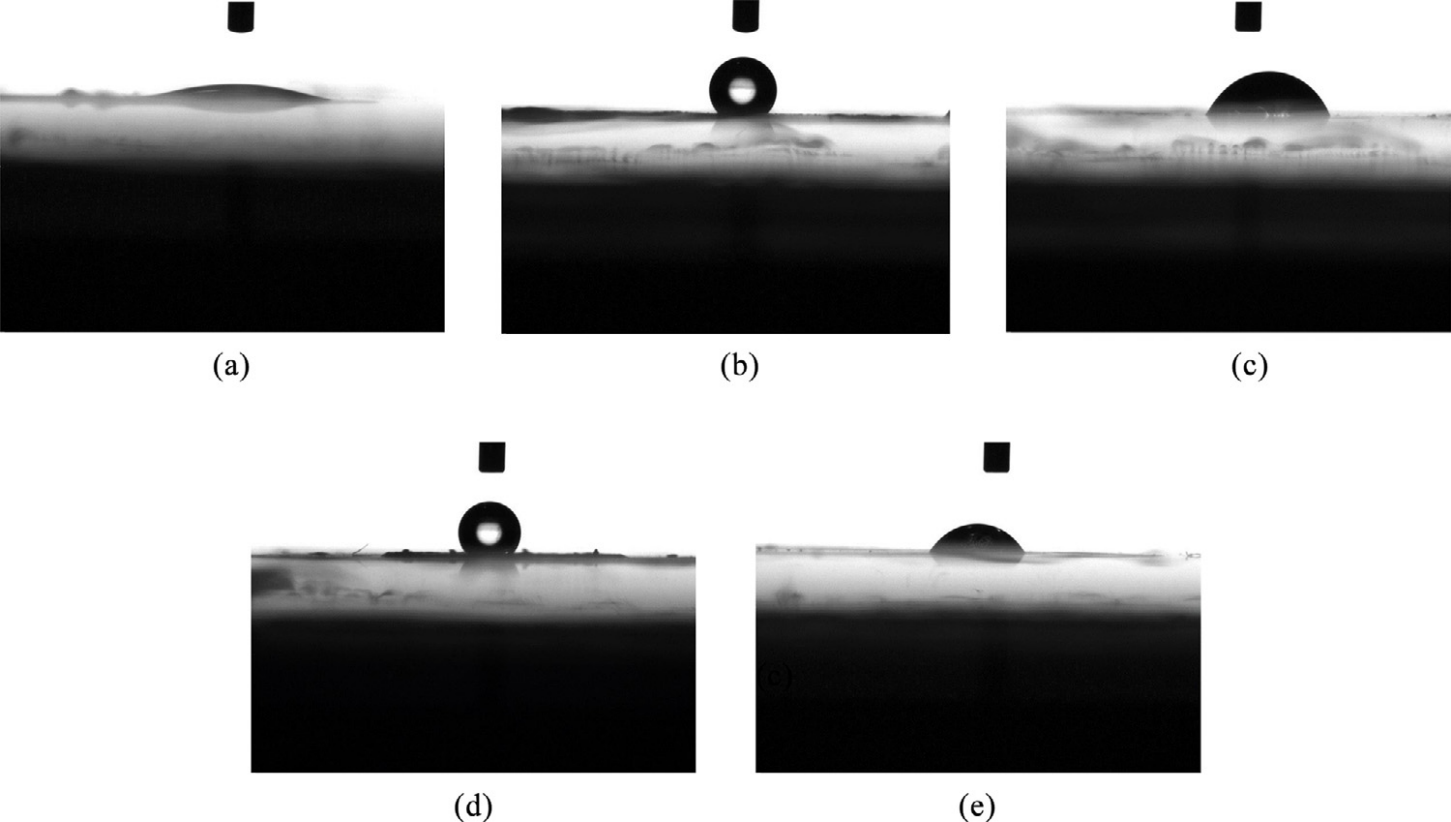
(A) A typical drop at the edge region of the microparticle film after the plasma treatment. (B) - A typical drop at the center region of the microparticle film before the plasma asher treatment. (C) - A typical drop at the edge region of the microparticle film before the plasma asher treatment. (D) - A typical drop at the center region of the microparticle film, following the plasma treatment and the mixing and redepositing of the particles. (E) - A typical drop at the edge region of the film, following the plasma treatment and the mixing and redepositing of the particles.

Figs. 5A-E show contact angle measurements of the microparticle films on glass, at different steps of preparation and at different regions of the film. In Fig. 5A, a hydrophilic angle is observed for a drop at the edge region of the film, following the plasma asher treatment. At the very center of the microparticle film, at this stage of preparation, a full wetting layer was achieved and no contact angle could be measured. Figs. 5B and 5C, respectively, show the contact angle measurements at the center and edge of the microparticle film, prior to the plasma asher treatment. According to these measurements, at this stage of the process the microparticle surface is hydrophobic, as can be expected due to the presence of the organic ligands. Contact angle measurements of films containing randomly oriented microparticles, prepared following the plasma treatment and a mixing of the particles, are presented in Figs. 5D and 5E for the center and edge of the film, respectively. The mixing and redeposition randomly expose the two opposite facets of the particles, yielding an intermediate contact angle at both the center and edge of the film.

Table 1 summarizes the results of the contact angle measurements. From the results it is clear that the pre-plasma particles are highly hydrophobic, while the plasma-treated particles are very hydrophilic. In the plasma-treated films, the multilayered center region was, in fact, so hydrophilic that it was not possible to measure the contact angle of the drop, and a full wetting of the surface was observed. After the mixing and redepositing of the particles at random orientation, intermediate values were obtained, indicating that the plasma did not affect the ligands facing the glass.

**Table. 1:** Contact angle values for microparticle films before the plasma treatment, after the plasma treatment, and after the plasma treatment and random re-orientation of the particles. The numbers in bracket represent the number of contact angle measurement repetitions performed on each type of sample.

	Before Plasma	After Plasma & mix	After Plasma
Center (multilayer)	128.0ᵒ ± 4.43ᵒ (6)	119.2ᵒ ± 3.04ᵒ (3)	Full wetting
Edge (monolayer)	55.3ᵒ ± 3.12ᵒ (4)	41.1ᵒ ± 13.53ᵒ (3)	13.6ᵒ ± 7.19ᵒ (4)

#### Applications

The resulting Janus magnetic particles take advantage of the CISS effect [2] to separate between chiral enantiomers. Microparticles fabricated using the procedure described here were utilized to create enantiomer separation filters [1]. Implementation of such particles into a separation column should serve to establish a generic method for enantio separation. Lastly, these particles are expected to be applicable for use in the field of non-symmetric catalysis.

## Declaration of Competing Interest

The authors declare that they have no known competing financial interests or personal relationships that could have appeared to influence the work reported in this paper.
